# A comprehensive dataset of protein-protein interactions and ligand binding pockets for advancing drug discovery

**DOI:** 10.1038/s41597-024-03233-z

**Published:** 2024-04-20

**Authors:** Alexandra Moine-Franel, Fabien Mareuil, Michael Nilges, Constantin Bogdan Ciambur, Olivier Sperandio

**Affiliations:** 1 Structural Bioinformatics Unit, Department of Structural Biology and Chemistry, Institut Pasteur, Université de Paris, CNRS UMR3528 Paris, France; 2 Collège Doctoral, Sorbonne Université, Paris, F-75005 France

**Keywords:** Target validation, Data mining

## Abstract

This dataset represents a collection of pocket-centric structural data related to protein-protein interactions (PPIs) and PPI-related ligand binding sites. The dataset includes high-quality structural information on more than 23,000 pockets, 3,700 proteins on more than 500 organisms, and nearly 3500 ligands that can aid researchers in the fields of bioinformatics, structural biology, and drug discovery. It encompasses a diverse set of PPI complexes with more than 1,700 unique protein families including some with associated ligands, enabling detailed investigations into molecular interactions at the atomic level. This article introduces an indispensable resource designed to unlock the full potential of PPIs while pioneering a novel metric for pocket similarity for hypothesizing protein partners repurposing.

## Background & Summary

Protein-protein interactions (PPIs) are the powerhouses of biological systems, managing a multitude of cellular tasks^1,2^. They hold central roles in the intricate processes of life. Unlocking the full potential of these Protein-Protein Interactions (PPIs), particularly through a pocket-centric approach^3–5^, is critical for comprehending cellular functions, diseases, and advancing drug discovery.

In this context, we introduce an innovative resource poised to transform biological research, with a primary focus on protein binding pockets. Our motivation for creating this dataset arises from the pressing need for a central, user-friendly repository that captures the essence of PPIs and ligand binding pockets for drug design purposes. This carefully curated dataset is designed to support researchers from various scientific fields, offering a rich source of structural insights, and leverages the concept of pocket similarity to infer potential protein partners.

### Importance of understanding PPIs and ligand binding pockets

PPIs are the dynamic partnerships that proteins form within a cell. They underpin vital processes such as signal transduction, DNA replication, and metabolic regulation. The specific binding of proteins to each other orchestrates these processes, making PPIs central to our comprehension of cell function. Ligand binding pockets, on the other hand, are molecular docking sites within proteins where various molecules, including small compounds and other proteins, bind. These interactions regulate protein function, control cellular responses, and are critical for drug discovery and targeted therapies.

### Motivation behind creating this dataset

**Fig. 1:**
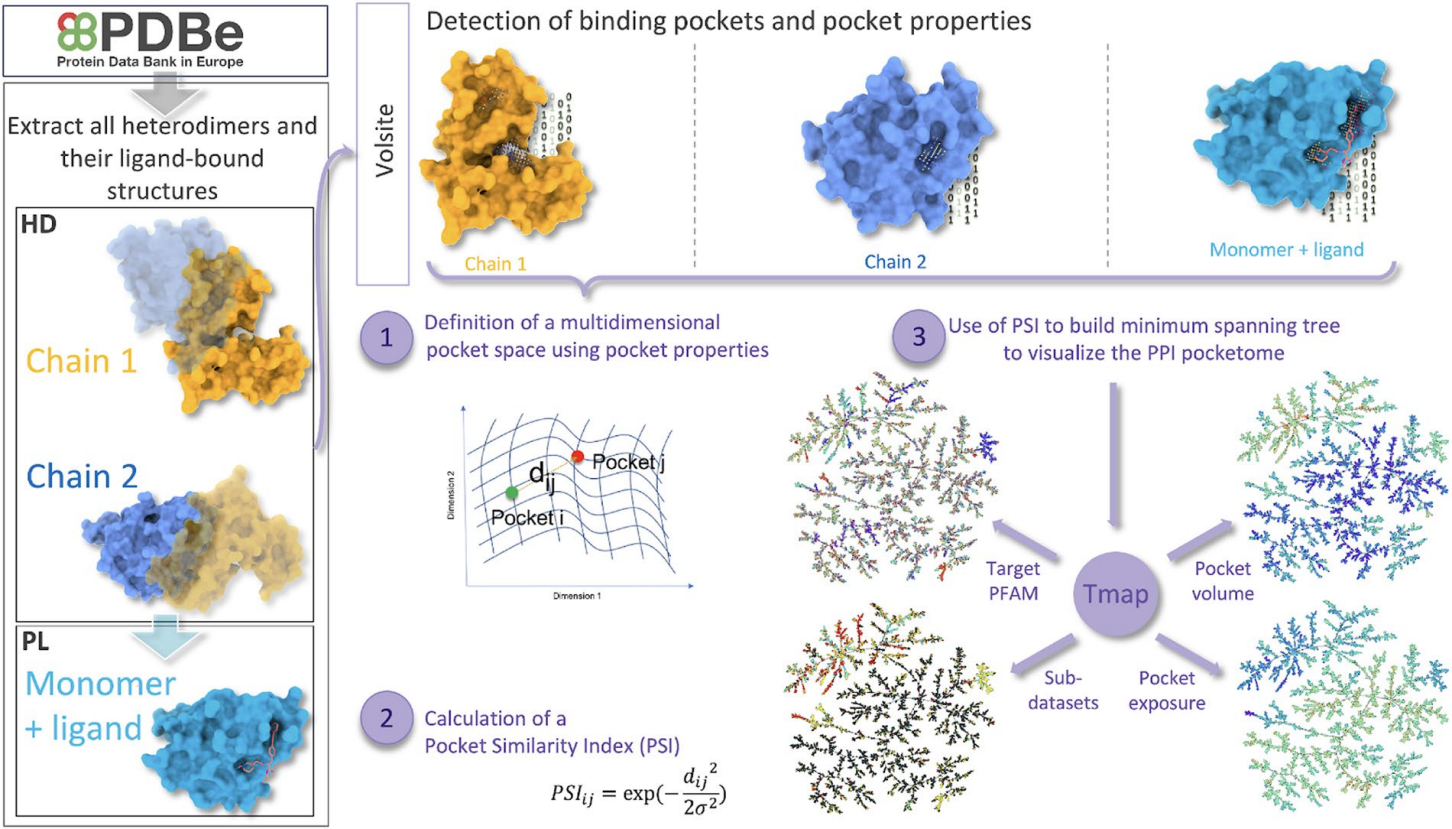
Description of the workflow used to build the dataset.

The motivation for creating the dataset presented in this article stems from the need for a comprehensive and accessible resource for researchers across diverse scientific domains. As a curated and structured repository of structural data pertaining to PPI complexes, ligands and ligand binding pockets (Fig. 1). The creation of such a dataset serves several essential purposes:

#### Advancing biological research

The dataset constitutes a centralized repository of structural data (more 23,000 pockets, 3,700 proteins on more than 500 organisms, 1,700 unique protein families, and nearly 3,500 ligands) on protein-protein interactions (PPIs) and ligand binding pockets, enabling researchers to conduct a wide range of studies in structural biology, bioinformatics, and systems biology. For instance, researchers can utilize the dataset to explore the structural basis of disease-associated PPIs, gaining insights into the molecular mechanisms underlying various pathological conditions. By analysing the interactions between proteins and their binding partners, researchers can identify potential therapeutic targets for drug intervention.

Furthermore, the dataset facilitates the investigation of molecular recognition and ligand binding specificity. Researchers can analyse the three-dimensional structures of PPI complexes and ligand binding pockets to elucidate the molecular determinants of binding affinity and selectivity. This knowledge is essential for understanding the molecular basis of drug action and designing drugs with enhanced potency and specificity.

#### Accelerating drug discovery

Understanding the structural characteristics of PPI complexes and ligand binding pockets is crucial for accelerating drug discovery efforts. The dataset provides detailed structural information on protein-ligand interactions, offering valuable insights into potential drug targets and binding sites. Researchers can leverage this information to identify druggable pockets within proteins and design small molecules or biologics that specifically target these sites.

Moreover, the dataset facilitates virtual screening and molecular docking studies to identify potential lead compounds for drug development. By computationally screening large libraries of compounds against the three-dimensional structures of target proteins on well profiled binding pockets, researchers can prioritize promising candidates for experimental validation. This accelerates the process of lead identification and optimization, ultimately expediting the development of novel therapeutics for various diseases, including cancer, infectious diseases, and neurological disorders.

### Enabling data-driven discoveries

Data scientists can use this dataset to uncover novel relationships between proteins, ligands, and their structural features, fuelling data-driven discoveries, hypothesis generation, and finally machine learning and AI.

### Development of a pocket similarity metric

One of the key highlights of our dataset is the development of a metric for pocket similarity. This metric allows for the comparison of the structural similarity of docking sites within proteins. Researchers can leverage this information to potentially repurpose protein partners based on structural commonalities, leading to novel insights and discoveries.

### Potential Applications in Scientific Research

The dataset presented here has wide-ranging applications in scientific research, including but not limited to:

- Elucidating the structural basis of disease-associated PPIs and identifying potential therapeutic targets.
- Investigating the mechanisms of molecular recognition and ligand binding specificity.
- Conducting large-scale computational analyses of PPI networks for systems biology studies.
- Supporting the development of personalized medicine by characterizing individual-specific interactions.

In summary, this dataset serves as a valuable resource that bridges the gap between fundamental molecular interactions and their practical applications in scientific research. It is our hope that researchers from various disciplines will find this dataset instrumental in their quest to unravel the mysteries of biology and develop innovative solutions for health and drug discovery.

## Methods

**Fig. 2:**
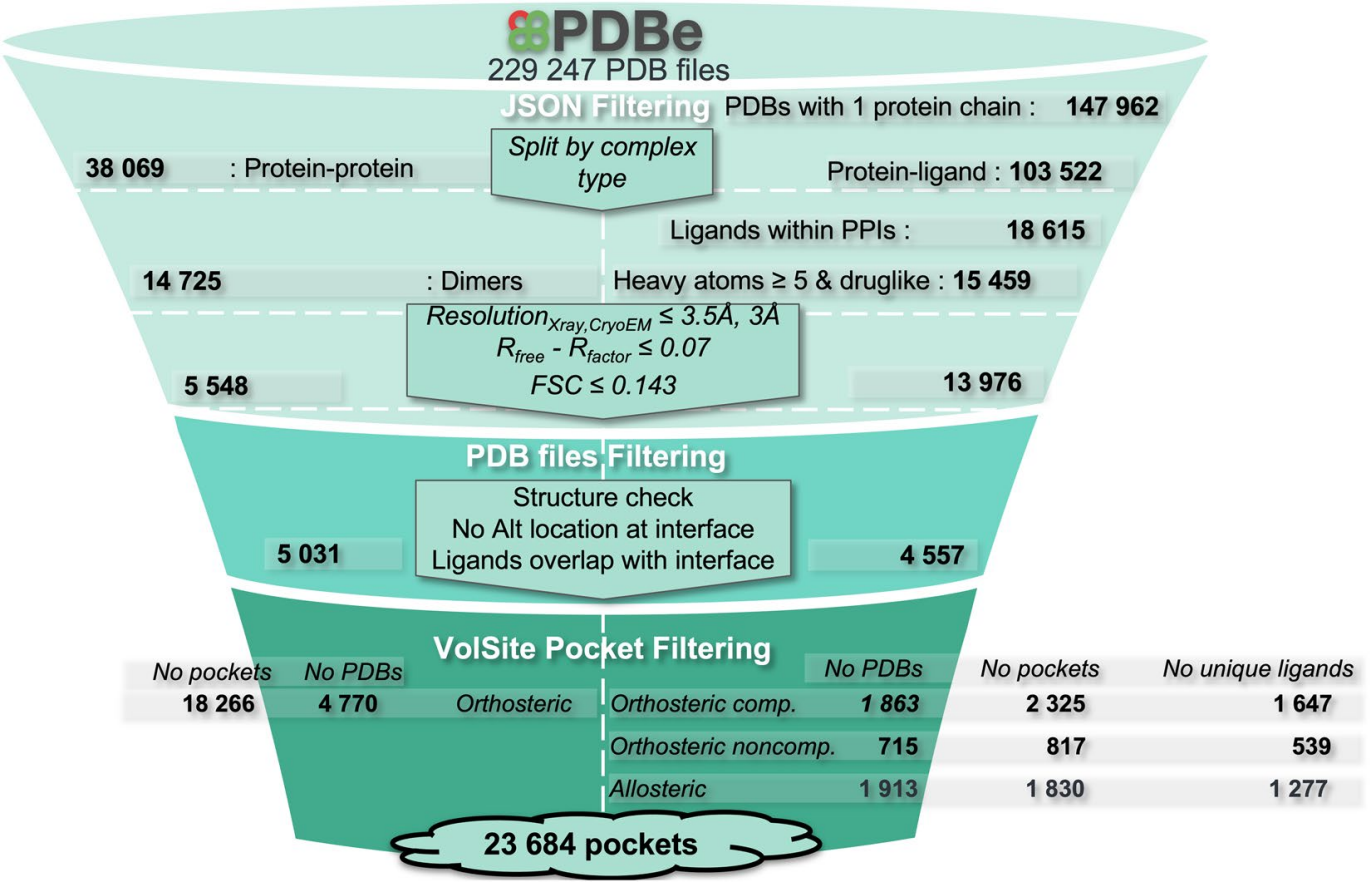
Full filtering protocol to prepare the datasets HD, PLOC, PLONC and PLA.

The preparation of the protein-protein dataset and the protein-ligand dataset involves several systematic steps to ensure data accuracy and relevance (Fig. 2).

### Protein selection

The preparation of the protein-protein dataset and the protein-ligand dataset involves several systematic steps to ensure data accuracy and relevance. Initially, the metadata of the entire PDB database^6^ (March 2023 version) is downloaded as a.json file from the protein databank in Europe (PDBe), serving as the primary data source. PDBe annotations and Uniprot^7^ identifiers (IDs) are then leveraged to identify two distinct subsets: heterodimers complexes (HD dataset) complexes, representing protein-protein interactions, and protein-ligand complexes. The next step involves cross-referencing the protein-ligand complexes with the HD dataset, ensuring that they share a Uniprot ID to only select protein-ligand pairs associated with one or several HD complexes (PL dataset). To refine the protein subset, we further impose the following rules: that HD complexes only contain two molecules with different Uniprot IDs, and each molecule has more than three residues. PL complexes are required to contain one molecule, and their ligands must have at least five heavy atoms, to ensure more complex ligands are considered. Moreover, ligands must exclusively consist of drug-like atoms, encompassing carbon (C), nitrogen (N), oxygen (O), sulphur (S), phosphorus (P), and halogens (X = I, Br, Cl, F), along with boron (B). In addition, certain ligands, such as ATP, co-factors and molecules originating from crystallization buffers were omitted from the list due to their limited relevance to drug design. The criterium used for this final step was based on the occurrence of the ligands (No of times < 10) in the PDB to list ligands to exclude followed by a visual inspection to ensure that we did not remove pertinent ligands.

Furthermore, following this preliminary curation, 3D structure quality filters were then applied to the subset. Only 3D structures determined by nuclear magnetic resonance, X-ray crystallography or cryogenic electron microscopy (cryo-EM) were selected. The resolution was imposed to be is lower or equal to 3.5 or 3 Å for X-ray and cryo-EM structures, respectively. The difference between the R-free and R-factor (for X-ray structures) and the Fourier shell correlation (for cryo-EM structures) was required to be lower or equal to 0.07 and 0.143, respectively. The list of PDB IDs requiring the download of the actual structure was then compiled and executed.

To enhance the dataset’s reliability, several post-download filtering steps were implemented. The 3D structures could not contain any atom with alternative locations at the protein-protein or protein-ligand interface. Moreover, the ligands of the PL datasets were sorted depending on their location with respect to an HD-associated interface. The definition of the interaction patch is based on the Euclidean distance between all atoms of the protein target and its partner. The distance threshold was fixed at 6 Angstroms (Å). This step ensures that the ligands are contextually relevant to the heterodimer complexes.

Before detecting pockets, incomplete amino acids in the structures were repaired by FoldX^8^ (version 5) software. Heteroatoms (only for HD complexes) and water molecules were removed and HD and PL complexes were ultimately protonated with the OPLS-AA force field of GROMACS^9^ (version 2020). Subsequently, the structures were converted into.mol2 format.

### Pockets detection, filtration and characterization

VolSite^10^ was employed to detect and characterize pockets. Pockets were detected within the monomers (PL) using the ligands as reference for the selection of surrounding residues for the VolSite pocket detection and profiling. Similarly, pockets were detected on one protein within the heterodimer (HD) with the other protein treated as the ligand, and vice versa, with the roles reversed. Given that pockets within PPIs typically exhibit distinct properties, such as shallowness, we adjusted the VolSite parameters to better suit the characteristics of PPI pockets using a selection of known liganded PPIs as a positive control (Table 1).

**Table 1:** List of VolSite parameters used to detect PPI pockets.

VolSite parameter	Description	Value
step	Edge length of each box (Å)	1
boxS	Edge length of the main box (Å)	20
b	Minimal threshold for buriedness	65
n	Minimal neighbours for buried cavity boxes	12
nPTS	Minimal number of cubes to consider it a cavity	20

In the context of heterodimer (HD) complexes, binding pockets were verified to reside at the interface, affirming their orthosteric nature. We delineated distinct types of ligand-binding pockets within protein-ligand complexes, differentiated into three main types based on their relationship with the protein-protein interaction (PPI) interface. These classifications are crucial not only for understanding the functional implications of ligand binding but also for training machine learning models to design focused chemical libraries.

**Fig. 3:**
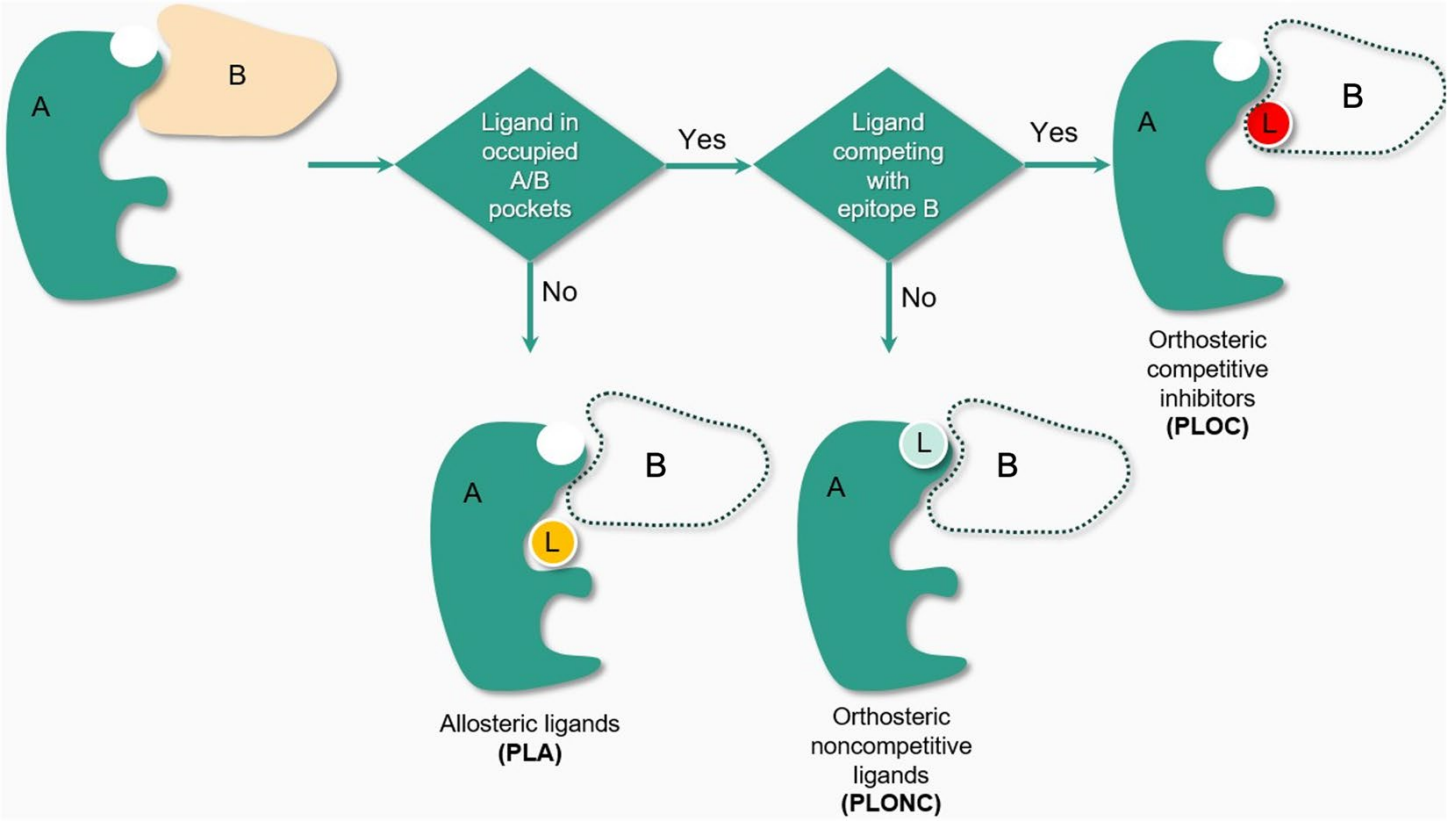
Creation of the Protein-Ligand (PL) datasets PLA, PLONC and PLOC.

We classified these pockets into three main types: orthosteric competitive (PLOC), orthosteric non-competitive (PLONC), and allosteric (PLA) pockets (Fig. 3). PLOC pockets involve direct competition between the ligand and the protein partner’s epitope within the heterodimer. In contrast, PLONC pockets house ligands within orthosteric pockets that don’t directly compete with the protein’s epitope but may influence its function or conformation. Finally, PLA pockets, situated near the orthosteric binding pockets of a heterodimer, don’t directly overlap with the orthosteric site but may induce allosteric effects.

Such classifications are crucial not only for understanding the functional implications of ligand binding but also for training machine learning models to design focused chemical libraries. The primary set of PL structures, comprising PLOC, could serve as positive datasets for machine learning models, while PLA could represent negative datasets for ligands binding to PPI-involved protein chains without direct interface proximity. Furthermore, the PLONC subset, capturing intermediate scenarios where ligands occupy pockets alongside protein epitopes, offers additional training data for nuanced scenarios. This dichotomy facilitates accurate discernment between competitive and non-competitive ligand interactions within protein-ligand complexes, aiding in the design of focused chemical libraries tailored to modulate protein-protein interactions.

To differentiate between PLOC and PLONC, we utilized a specific criterion: pockets where the ligand was located within 1 Å of their protein partner were labelled as PLOC, while those with ligands positioned more than 1 Å away were annotated as PLONC. While acknowledging the potential limitations of this approach, such as ligands inducing conformational changes affecting the PPI surface, we recognize the complexity involved and view our approach as a foundational step in classification.

### Definition of a PPI pocketome and of a pocket similarity index (PSI)

To assess the similarity between different pockets, we developed a metric that relies on pocket properties (e.g. volume, exposure, asphericity, etc…) as encoded by a set of quantitative descriptors. The VolSite software was utilized to calculate an extensive initial array of 89 pocket descriptors. Then, a set of 10 supplementary descriptors amalgamating attributes from those initially derived by VolSite was computed, to furnish a more nuanced depiction of pocket characteristics. These 10 amalgamated descriptors were determined in accordance with the methodology outlined in Kuenemann *et al*. in 2016^11^.

**Fig. 4:**
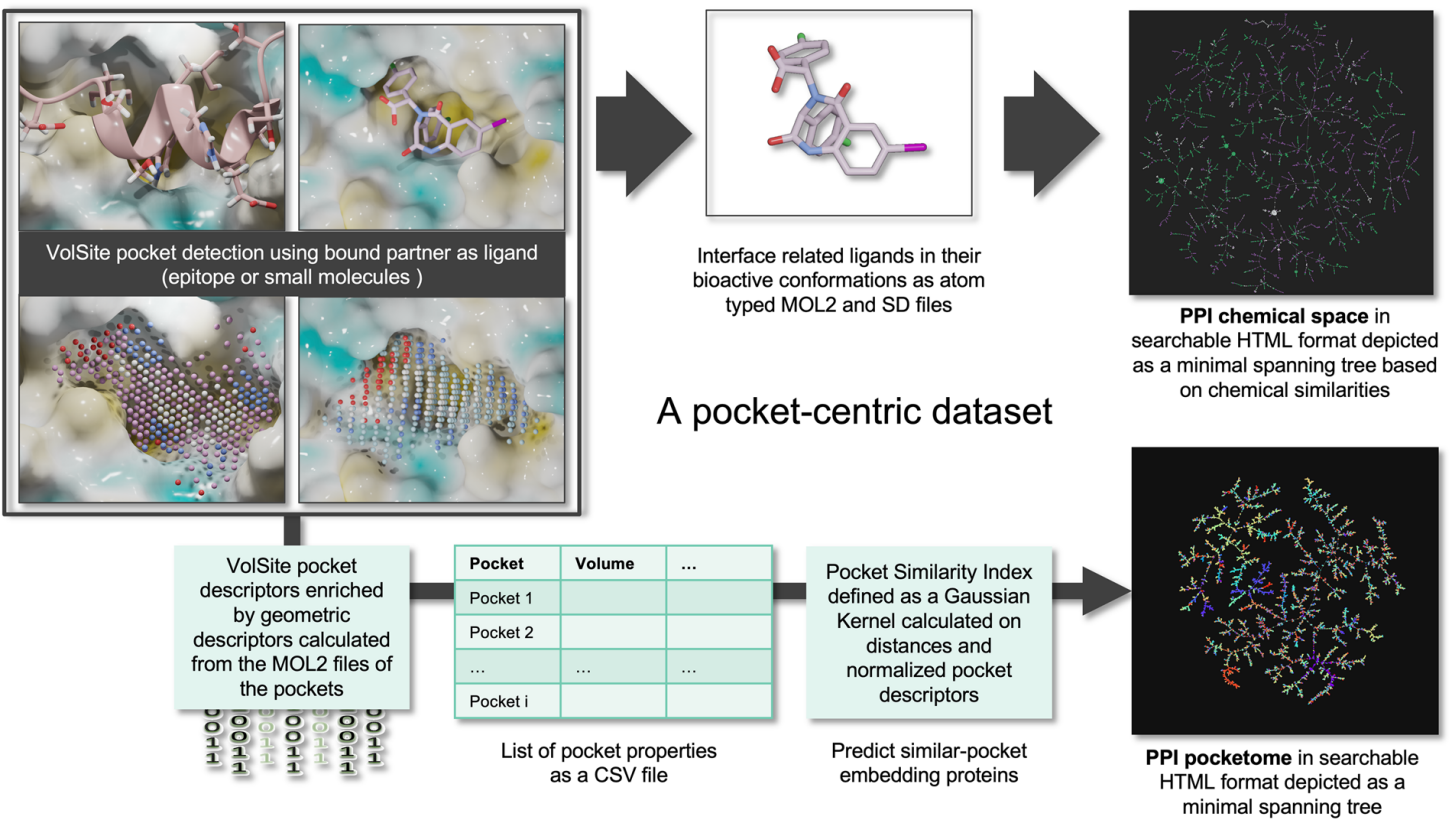
Creation of a PPI pocketome using pocket descriptor and a pocket similarity index (PSI).

Finally, 10 more geometric descriptors were computed using the RDKit3D module, to encompass key pocket properties such as asphericity, sphericity index, molecular eccentricity, inertial shape factor, radius of gyration, principal moments of inertia, and normalized principal moments ratio. To keep consistency, these latter descriptors were specifically calculated using the MOL2 files of the VolSite pockets (Fig. 4).

From the total of 109 pocket descriptors obtained above we discarded those with zero values for more than 95% of the pockets in our sample, reducing the number to 82 descriptors. The distributions of their descriptor values were subsequently analysed to ensure that each descriptor provides an equitable and non-redundant contribution towards the similarity metric. Descriptors with a high dynamic range and highly skewed distributions were re-scaled to a logarithmic scale, specifically if their distribution satisfied the following conditions: *(i.)* the mean value <15% of the maximum value, and *(ii.)* the median <65% of the mean. Finally, the descriptors were all re-scaled to have zero mean and unit variance, to minimise bias or any covariance.

The similarity between two pockets was evaluated based on their distance in this resulting high-dimensional space of pocket descriptors. Thus, the Euclidean distance was calculated pairwise for the entire sample, resulting in a N × N distance matrix, with N = 23238 pockets. A Gaussian kernel was then applied to this matrix to transform the distance values into probabilities between 0 and 1, constituting what we define as the *pocket similarity index* (PSI):

1 $$PS{I}_{ij}=\exp \left(\frac{-{d}_{ij}^{2}}{2{\sigma }^{2}}\right)$$

where *d*_*ij*_ is the Euclidean distance between pockets *i* and *j* (*i ≠ j*), and *σ* is the standard deviation of *d* across all pairs.

The pocketome is visualised as a minimum spanning tree using the tool TMAP^12^. This method selects only certain pairs from the complete PSI matrix in such a way as to span the entire dataset (no point is disconnected) in the optimal way, i.e. by minimising the *total* distance. The resulting “tree” therefore is a simplified yet powerful visual representation of our entire dataset, built to reflect local proximity (so pocket similarity) in the high dimensional pocketome space. With this tool, provided in interactive *html* format, users can quickly assess local clustering of similar pockets, but also study trends in the data with a number of pocket properties, such as their volume, exposure, asphericity, HD/PL or Pfam annotation, etc. We refer the reader to the Technical Validation section below for further details.

Through this meticulous process of data collection, filtering, and classification, the resulting datasets are refined and enriched, facilitating comprehensive research on protein-protein interactions and ligand binding pockets, with potential implications for drug discovery. Specifically, by amalgamating a set of liganded binding pockets with an exhaustive catalogue of heterodimer pockets, we aim to create a resource intended to serve as a robust platform for identifying new potential protein-protein interaction (PPI) drug targets. This combined dataset not only enhances our understanding of protein interactions but also provides researchers with a valuable tool to explore novel avenues for drug development, potentially leading to the discovery of innovative therapeutic interventions (Fig. 4).

## Data Records

The dataset is openly available^13^ with the 10.5281/zenodo.10805580 under a CC BY 4.0 licence.

This detailed dataset provides a comprehensive view of protein binding pockets, offering geometric, structural, and classification information crucial for understanding their properties and potential functional roles.

In the following, we describe in detail one example of an HD system. For an HD system the provided folders and files always follows the same pattern. This example corresponds to the PDB code 1bxl, representing a heterodimer (HD) composed of two chains. Specifically, chain A denotes Bcl-2-like protein 1 (UniProt ID: Q07817) in complex with chain B, which represents Bcl-2 homologous antagonist/killer (UniProt ID: Q16611).

- 1bxl--AB--Q07817--Q16611.pdb: PDB file representing the heterodimeric complex of proteins Q07817 and Q16611 within the 1bxl structure.
- 1bxl--A--Q07817__Repair-H.pdb: PDB file representing the protein Q07817 (chain A) after the repair process by FoldX and protonation.
- 1bxl--B--Q16611__Repair-H.pdb: PDB file representing the protein Q16611 (chain B) after the repair process by FoldX and protonation.
- pdb1bxl.ent: Raw PDB file without any processing or modifications.
- results/1bxl-AB-Q07817-Q16611-withinA:
- 1bxl-AB-Q07817-Q16611-withinA_CAVITY_N1_ALL_orthosteric.mol2: Mol2 file describing the orthosteric VolSite pocket (CAVITY_N1) of the heterodimeric complex within chain A.
- 1bxl-AB-Q07817-Q16611-withinA_CAVITY_N2_ALL_orthosteric.mol2: Mol2 file describing another orthosteric VolSite pocket (CAVITY_N2) within chain A.
- 1bxl-AB-Q07817-Q16611-withinA_CAVITY_N3_ALL_nonorthosteric.mol2: Mol2 file representing a non-orthosteric VolSite pocket (CAVITY_N3) within chain A.
- 1bxl-AB-Q07817-Q16611-withinA_CAVITY_N4_ALL_nonorthosteric.mol2: Mol2 file representing another non-orthosteric VolSite pocket (CAVITY_N4) within chain A.
- 1bxl-AB-Q07817-Q16611-withinA_CAVITY_N5_ALL_nonorthosteric.mol2: Mol2 file representing a non-orthosteric VolSite pocket (CAVITY_N5) within chain A.
- 1bxl-AB-Q07817-Q16611-withinA_CAVITY_N6_ALL_nonorthosteric.mol2: Mol2 file representing a non-orthosteric VolSite pocket (CAVITY_N6) within chain A.
- 1bxl-AB-Q07817-Q16611-withinA_CAVITY_N7_ALL_nonorthosteric.mol2: Mol2 file representing a non-orthosteric VolSite pocket (CAVITY_N7) within chain A.
- 1bxl-AB-Q07817-Q16611-withinA_CAVITY_N1_ALL__.mol2: Mol2 file representing the orthosteric VolSite pocket (CAVITY_N1) without specific ligand information.
- 1bxl-AB-Q07817-Q16611-withinA_CAVITY_N2_ALL__.mol2: Mol2 file representing the orthosteric VolSite pocket (CAVITY_N2) without specific ligand information.
- 1bxl-AB-Q07817-Q16611-withinA_CAVITY_N3_ALL__.mol2: Mol2 file representing a non-orthosteric VolSite pocket (CAVITY_N3) without specific ligand information.
- 1bxl-AB-Q07817-Q16611-withinA_CAVITY_N4_ALL__.mol2: Mol2 file representing a non-orthosteric VolSite pocket (CAVITY_N4) without specific ligand information.
- 1bxl-AB-Q07817-Q16611-withinA_CAVITY_N5_ALL__.mol2: Mol2 file representing a non-orthosteric VolSite pocket (CAVITY_N5) without specific ligand information.
- 1bxl-AB-Q07817-Q16611-withinA_CAVITY_N6_ALL__.mol2: Mol2 file representing a non-orthosteric VolSite pocket (CAVITY_N6) without specific ligand information.
- 1bxl-AB-Q07817-Q16611-withinA_CAVITY_N7_ALL__.mol2: Mol2 file representing a non-orthosteric VolSite pocket (CAVITY_N7) without specific ligand information.
- state.log, state_step5.log: Log files containing information about the state or progress of the processing steps.
- results/1bxl-BA-Q16611-Q07817-withinB:
- 1bxl--B--Q16611__Repair-H_descriptors_3d.csv: CSV file containing 3D descriptors for the repaired structure of protein Q16611 (chain B) within chain B.
- state.log: Log file containing information about the state or progress of the processing steps.
  These files and directories provide a detailed view of the heterodimeric interactions between proteins Q07817 and Q16611, including orthosteric and non-orthosteric binding sites and descriptors essential for understanding the binding characteristics within the 1bxl structure.
  Below is the description of one example of a PL system. For an PL system the provided folders and files always follows the same pattern. This example corresponds to the PDB code 1t4e, representing a PLOC PL structure composed of one chain and one ligand. Specifically, chain A denotes E3 ubiquitin-protein ligase Mdm2 (Uniprot ID – Q00987) in complex with ligand DIZ.
- 1t4e_A_DIZ_112.mol2: Mol2 file containing the ligand in its bioactive conformation, with detailed molecular information, such as atom types and bond properties, specifically for the orthosteric competitive binding site.
- 1t4e_A_DIZ_112.sdf: SDF (Structure Data File) format containing the ligand in its bioactive conformation for the orthosteric competitive binding site, including molecular properties.
- 1t4e--A--Q00987--DIZ-112__2mps--A--Q00987__aligned.pdb: PDB file where the protein structure (1t4e) from chain A is superimposed onto the corresponding heterodimer HD (2mps) structure, with the ligand (DIZ-112) indicated as an inhibitor.
- 1t4e--A--Q00987--DIZ-112__interface-residues_6A.txt: Text file containing a list of residues in contact with the ligand (DIZ-112) in the 1t4e structure, within a 6 Å distance threshold.
- 1t4e--A--Q00987–DIZ-112_orthosteric_competitive.pdb: PDB file representing the orthosteric competitive binding site of the protein (1t4e) with the ligand (DIZ-112) in its bioactive conformation.
- 1t4e--A--Q00987.pdb: PDB file representing chain A of the protein structure 1t4e, which is part of the heterodimer.
- pdb1t4e.ent: Raw PDB file without any processing or modifications.
- results/1t4e-A-Q00987-DIZ-112: Directory containing various files related to the binding pockets and ligands within the 1t4e protein structure.
- 1t4e-A-Q00987-DIZ-112_CAVITY_N1_ALL_liganded_orthosteric_competitive.mol2: Mol2 file describing the liganded orthosteric competitive VolSite pocket (CAVITY_N1) within the 1t4e structure.
- 1t4e-A-Q00987-DIZ-112_CAVITY_N2_ALL_unliganded.mol2: Mol2 file representing the unliganded VolSite pocket (CAVITY_N2) within the 1t4e structure.
- 1t4e--A--Q00987__Repair-H_descriptors_3d.csv: CSV file containing 3D descriptors for the repaired structure.
- CAVITY_N1_ALL__.mol2: Mol2 file describing the orthosteric competitive VolSite pocket (CAVITY_N1) without specific ligand information.
- CAVITY_N2_ALL__.mol2: Mol2 file representing an unliganded VolSite pocket (CAVITY_N2) without specific ligand information.
- state.log, state_step5.log: Log files containing information about the state or progress of the processing steps.
  This detailed dataset provides a comprehensive view of protein-ligand interactions, including ligand structures, protein-ligand complexes, binding pocket information, and related descriptors. The files are meticulously organized and annotated, facilitating in-depth analysis and understanding of the protein-ligand interactions within the specified structures.
  Several csv files are provided as collections of annotated binding pockets:
- HD_part8_20230317_matrix_orthosteric.csv
- HD_part8_20230317PDBe_orthosteric__complete.csv
- PL_part8_20230317_matrix_liganded_allosteric.csv
- PL_part8_20230317PDBe_allosteric__complete.csv
- PL_part8_20230317_matrix_liganded_orthosteric_competitive.csv
- PL_part8_20230317PDBe_orthosteric_competitive__complete.csv
- PL_part8_20230317_matrix_liganded_orthosteric_noncompetitive.csv
- PL_part8_20230317PDBe_orthosteric_noncompetitive__complete.csv
  The provided CSV file contains a wealth of data pertaining to protein binding pockets, including VolSite pocket properties, geometric attributes derived from the negative image of the VolSite pockets stored in Mol2 format. The CSV files with the name containing “_complete” represent subsets of the former files and contain supplementary annotations related to CATH and PFAM classifications. Below is a description of the columns in the CSV file:
- cath: CATH classification of the protein domain to which the pocket belongs (only in csv file names containing “__complete”).
- pdb.chain: Identifier for the protein chain in the PDB structure.
- Cavity: Identifier for the specific pocket or cavity on the protein chain.
  - ex:4lgu-A-Q13490-1YH-402_CAVITY_N2_liganded_orthosteric_competitive

- Pocket is found in pdb 4glu
- within chain A
- the Uniprot ID of chain A is Q13490
- Ligand ID is 1YH
- Ligand residue number is 402
- Volsite pocket is N2
- the ligand found in this pocket is classified as ligand orthosteric competitive

- Volume: Volume of the pocket, indicating its size.
- CZ, CA, O, OD1, OG, N, NZ, DU: VolSite pockets properties based on polarity such aromaticity, hydrophobicity, positively/negatively charged etc.
- CZ40, CZ40.50,…, NZ120: VolSite pockets properties by thresholds of pocket probe burial from 40 (exposed) to 120 (buried) geometric properties calculated from the negative image of the pocket.
- T40, T40.50, …, T110.120: Combination of VolSite properties that aggregates the burial metric of the pocket regardless of the polarity of the probe.
- PMI1, PMI2, PMI3: Principal moments of inertia, describing the pocket’s shape derived from the negative image of the VolSite pockets stored in Mol2 format.
- NPR1, NPR2: Normalized polar requirement values derived from the negative image of the VolSite pockets stored in Mol2 format.
- Rgyr: Radius of gyration, a measure of the pocket’s compactness derived from the negative image of the VolSite pockets stored in Mol2 format.
- Asphericity: Measure of deviation from a perfect sphere derived from the negative image of the VolSite pockets stored in Mol2 format.
- SpherocityIndex: Index indicating how spherical the pocket is, derived from the negative image of the VolSite pockets stored in Mol2 format.
- Eccentricity: Measure of the pocket’s elongation, derived from the negative image of the VolSite pockets stored in Mol2 format.
- InertialShapeFactor: Measure of the pocket’s mass distribution, derived from the negative image of the VolSite pockets stored in Mol2 format.
- pdb_code_index: Identifier for the PDB code corresponding to the protein structure.
- chain_index: Identifier for the chain within the PDB structure.
- pfam_accession: PFAM accession number, indicating the protein family to which the pocket-embedding protein belongs. (only in csv file names containing “__complete”)
- pfam_name: Name of the PFAM protein family (only in csv file names containing “__complete”).
- class, architecture, topology: CATH hierarchical classifications describing the protein domain (only in csv file names containing “__complete”).
- homologous: Indicates whether the pocket-embedding protein has homologous structures (only in csv file names containing “__complete”).
- cath_name: CATH classification name of the protein domain (only in csv file names containing “__complete”).

## Technical Validation

### Data collection and filtering

In meticulously curating our dataset, rigorous filtration processes were implemented to ensure its quality and relevance. After all filtering steps, four distinct subsets were built HD, PLOC, PLONC, and PLA (Table 2).

**Table 2:** Summary of the four HDPL subsets.

Datasets	No PDBs	No Pockets	No Pfam	No Uniprot	No Unique ligands	Metadata coverage
HD	4770	18266	821	1735	NA	57%
PLOC	1863	2325	76	119	1647	69%
PLONC	715	817	42	71	539	53%
PLA	1613	1830	83	112	1277	72%

1. HD Dataset:

- The HD (Hetero-Dimer) dataset represents a comprehensive collection of proteins with diverse Pfam^14^ domains. These proteins are involved in a wide range of biological processes and are characterized by a high level of functional variety.

2. PLOC Subset:

- The PLOC (Protein-Ligand Orthosteric Competitive) subset comprises proteins from the HD dataset that interact with ligands through orthosteric binding sites. Orthosteric ligands compete with native protein partners, modulating protein activity. Proteins in this subset are involved in various cellular processes and play roles in ligand binding, signal transduction, and regulation of metabolic pathways.

3. PLONC Subset:

- The PLONC (Protein-Ligand Orthosteric Non-Competitive) subset comprises proteins from the HD dataset that interact with ligands through orthosteric binding sites. These ligands modulate protein function remotely by binding to a common epitope pocket without directly competing with native protein partners. Proteins in this subset are associated with diverse biological functions, including enzyme regulation, receptor signalling, and cellular transport.

4. PLA Subset:

- The PLA (Protein-Ligand Allosteric) subset consists of proteins in the HD dataset that interact with ligands at allosteric binding sites. Allosteric ligands bind to sites distinct from the interaction site. Proteins in this subset are involved in complex regulatory networks, including enzyme regulation, signal transduction, and gene expression control, and comprises a fair amount of protein kinases.

### Metadata: protein fold and pfam domains

**Fig. 5:**
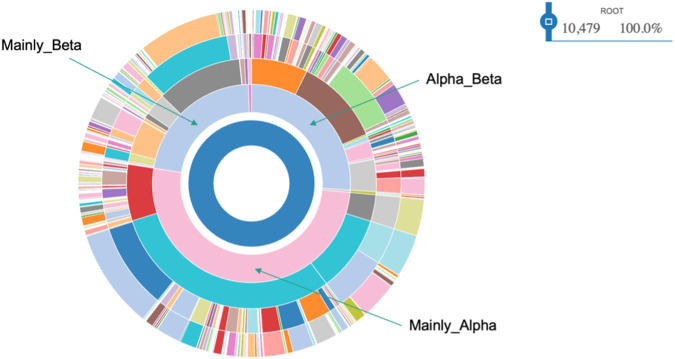
CATH classification of the pocket-embedding protein chains of the HD dataset.

We could fetch the metadata for a fair amount of the detected pocket-embedding protein chains ranging from 53% for PLONC to 72% for PLA. Those metadata consist of CATH architectures and Pfam name and IDs. We could check for the 10,479 pockets of the HD for which metadata were available, that the 3 main classes (Mainly_Beta, Main_Alpha, Alpha_Beta) of the CATH classification of protein 3D-folds are well represented in the HD dataset (Fig. 5). A similar trend was also true for the 3 PL subsets.

The HD dataset contains a large variety of heterodimers with various cellular functions including the ones that have been investigated for drug design purposes (Table 2).

**Fig. 6:**
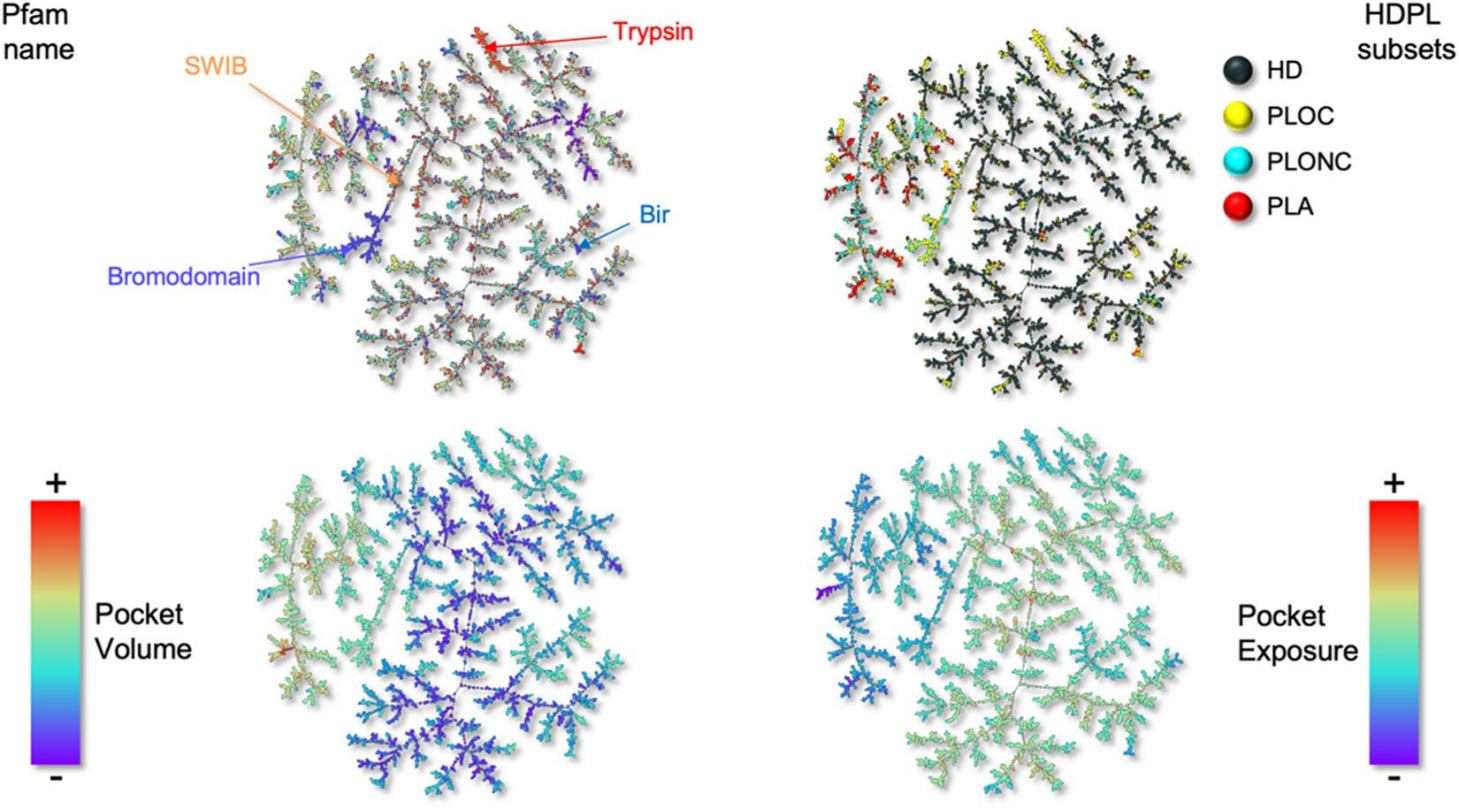
Pocketome represented as a minimum spanning tree. Each pocket is represented as a node in the tree and the proximity of pockets in the tree highlight their similarity. TMAP coloured by Pfam (top left panel), by HDPL subsets (top right), by pocket volume (bottom left), and finally by pocket exposure (bottom right).

A significant contribution of this study is the introduction of a straightforward metric, termed PSI (Pocket Similarity Index), designed to assess pocket similarities within the multidimensional pocketome space. This enables the classification, clustering, and inference of protein partner repurposing. Utilizing the PSI in conjunction with the minimum spanning tree method developed by Probst *et al*.^12^, we constructed a tree representing the pocketome of the HDPL datasets. Each pocket is depicted as a node in the tree, and edges connect pockets displaying a sufficiently high PSI. Notably, the tree incorporates the number of shared neighbours to establish connections, enhancing its accuracy. The resulting TMAP, available as an.html file, offers a valuable tool for pocketome analysis and exploration. Additionally, we employed the TMAP to colour-code the binding pockets based on various properties, including pocket volume, exposure, different subsets, and Pfam names (Fig. 6), providing a comprehensive visual representation of the intricate pocketome landscape.

The technical validation of our metric, in conjunction with TMAP, was first conducted by colour-coding pockets in the tree based on their Pfam annotations. This highlighted a strong alignment of Pfam names with the local branches of the tree, demonstrating the robustness and accuracy of our approach. Furthermore, when we applied colour-coding to differentiate between datasets (HD, PLOC, PLONC, and PLA) in the tree, we observed consistent branch homogeneity and distinct separation among subsets. Notably, certain branches contained pockets from multiple subsets, notably unliganded HD pockets in close proximity to successfully liganded pockets. Additionally, the colour-coding based on pocket properties, such as volume and exposure, provided further confirmation of our earlier observations, particularly the trend of HD pockets being smaller and shallower compared to those in the PL subsets. These findings validate the reliability and versatility of our metric and its integration with TMAPs, offering valuable insights into the structural intricacies of protein pockets.

### Supplementary information

Rejection files

## Data Availability

No custom code was used during this study for the curation and/or validation of the dataset.

## References

[1] Keskin O, Tuncbag N, Gursoy A (2008). Characterization and prediction of protein interfaces to infer protein-protein interaction networks. Curr Pharm Biotechnol.

[2] Gokhale A, Weldeghiorghis TK, Taneja V, Satyanarayanajois SD (2011). Conformationally constrained peptides from CD2 to modulate protein-protein interactions between CD2 and CD58. J Med Chem.

[3] Meireles LMC, Dömling AS, Camacho CJ (2010). ANCHOR: A web server and database for analysis of protein-protein interaction binding pockets for drug discovery. Nucleic Acids Res.

[4] Koes DR, Camacho CJ (2012). PocketQuery: Protein-protein interaction inhibitor starting points from protein-protein interaction structure. Nucleic Acids Res.

[5] Kumar V, Mahato S, Munshi A, Kulharia M (2018). PPInS: a repository of protein-protein interaction sitesbase. Sci Rep.

[6] Berman HM (2000). The Protein Data Bank. Nucleic Acids Res.

[7] Bateman A (2023). UniProt: the Universal Protein Knowledgebase in 2023. Nucleic Acids Res.

[8] Schymkowitz, J. *et al*. The FoldX web server: an online force field. *Nucleic Acids Res* **33**, (2005). 10.1093/nar/gki387 PMC1160148 15980494

[9] Van Der Spoel D (2005). GROMACS: Fast, flexible, and free. J Comput Chem.

[10] Desaphy J, Azdimousa K, Kellenberger E, Rognan D (2012). Comparison and druggability prediction of protein-ligand binding sites from pharmacophore-annotated cavity shapes. J Chem Inf Model.

[11] Kuenemann MA, Labbé CM, Cerdan AH, Sperandio O (2016). Imbalance in chemical space: How to facilitate the identification of protein-protein interaction inhibitors. Sci Rep.

[12] Probst D, Reymond JL (2020). Visualization of very large high-dimensional data sets as minimum spanning trees. J Cheminform.

[13] (2023). Zenodo.

[14] Mistry J (2021). Pfam: The protein families database in 2021. Nucleic Acids Res.

