# Supplementary material for: A comprehensive dataset of protein-protein interactions and ligand binding pockets for advancing drug discovery
Source: Sci Data. 2024 Apr 20;11:402. doi: 10.1038/s41597-024-03233-z (PMC11032347; doi:10.1038/s41597-024-03233-z)
Supplement: Supplementary file 1 — Rejection files [file 41597_2024_3233_MOESM1_ESM.docx]

Here is a list of txt files that contain the reason for the rejection of a given structure:

PART 1:

- 27,016 without ligand
- 17,424 without wanted ligand
- 2,889 without UniProt annotation
- 2,763 without ligand with heavy atoms (threshold = 5)
- 393 without ligand with drug-like element
- 14 without experimental method selected for ligand (as of 2023-03-17)
- 21 without experimental method annotation for ligand (as of 2023-03-17)

12 without experimental method selected for heterodimer (as of 2023-03-17)

- 6 without experimental method annotation for heterodimer (as of 2023-03-17)
- 68 without X-ray resolution for heterodimer (as of 2023-03-17)
- 54 without X-ray resolution for ligand (as of 2023-03-17)
- 165 without X-ray resolution factor for heterodimer (as of 2023-03-17)
- 567 without X-ray resolution factor for ligand (as of 2023-03-17)
- 308 without cryo-resolution for heterodimer (as of 2023-03-17)
- 228 without cryo-resolution for ligand (as of 2023-03-17)
- 3 without cryo-resolution cutoff for heterodimer (as of 2023-03-17)
- 1 without cryo-resolution cutoff for ligand (as of 2023-03-17)

PART 2:

- 13 without PDB structure for heterodimer (as of 2023-03-17)
- 165 without PDB structure for ligand (as of 2023-03-17)
- 0 without UniProt annotation for heterodimer (as of 2023-03-17)
- 3 without two molecules in heterodimer (as of 2023-03-17)
- 30 without protein-protein interactions in heterodimer (as of 2023-03-17)
- 46 without only one molecule in ligand (as of 2023-03-17)
- 8,313 without interface overlap for ligand (as of 2023-03-17)

PART 3:

- 1,422 with alternative locations at interface in heterodimer (as of 2023-03-17)
- 1,664 with alternative locations at interface in ligand (as of 2023-03-17)

PART 4:

- 97 without liganded pocket for ligand
- 208 without orthosteric pocket in heterodimer (as of 2023-03-17)

PART 5:

- 141 without corresponding UniProt data (part 6)
- 1,874 without orthosteric ligand (as of 2023-03-17, part 6)

PART 6:

- 494 without orthosteric ligand at minimum distance (as of 2023-03-17)
